# Phosphorus removal in denitrifying woodchip bioreactors varies by wood type and water chemistry

**DOI:** 10.1007/s11356-021-15835-w

**Published:** 2021-08-30

**Authors:** Ana Paula Sanchez Bustamante-Bailon, Andrew Margenot, Richard A. C. Cooke, Laura E. Christianson

**Affiliations:** 1 Department of Crop Sciences, University of Illinois at Urbana-Champaign, AW-101 Turner Hall, 1103 S. Goodwin Ave, Urbana, IL 61801 USA; 2 Department of Agricultural and Biological Engineering, University of Illinois at Urbana-Champaign, 1304 W. Pennsylvania Ave, Urbana, IL 61801 USA

**Keywords:** Batch test, Dissolved phosphorus, Oak, Poplar, Precipitation, Sorption

## Abstract

Denitrifying woodchip bioreactors are a practical nitrogen (N) mitigation technology but evaluating the potential for bioreactor phosphorus (P) removal is highly relevant given that (1) agricultural runoff often contains N and P, (2) very low P concentrations cause eutrophication, and (3) there are few options for removing dissolved P once it is in runoff. A series of batch tests evaluated P removal by woodchips that naturally contained a range of metals known to sorb P and then three design and environmental factors (water matrix, particle size, initial dissolved reactive phosphorus (DRP) concentration). Woodchips with the highest aluminum and iron content provided the most dissolved P removal (13±2.5 mg DRP removed/kg woodchip). However, poplar woodchips, which had low metals content, provided the second highest removal (12±0.4 mg/kg) when they were tested with P-dosed river water which had a relatively complex water matrix. Chemical P sorption due to woodchip elements may be possible, but it is likely one of a variety of P removal mechanisms in real-world bioreactor settings. Scaling the results indicated bioreactors could remove 0.40 to 13 g DRP/ha. Woodchip bioreactor dissolved P removal will likely be small in magnitude, but any such contribution is an added-value benefit of this denitrifying technology.

**Supplementary Information:**

The online version contains supplementary material available at 10.1007/s11356-021-15835-w.

## Introduction

Denitrifying woodchip bioreactors, woodchip-filled trenches where maintenance of anoxic conditions enhance denitrification, are a simple on-farm technology promoted for nitrate-nitrogen (NO_3_-N) treatment in agricultural drainage waters and effluents worldwide (Schipper et al. 2010). However, many areas impacted by non-point source N pollution also suffer from phosphorus (P)-related water quality impairment. Even though woodchip bioreactors leach nutrients, including P, upon start-up (Bell et al. 2015; Cameron and Schipper 2010; Healy et al. 2012), there have also been reports of P removal by bioreactors although the mechanisms and fate of the P are unclear (Husk et al. 2018). Woodchips can obviously act as a physical filter to trap sediment and particulate P (Choudhury et al. 2016; Sharrer et al. 2016), but beyond this, woodchip bioreactors have provided dissolved reactive P (DRP) removal ranging from 0.01 to 0.88 g DRP/m^3^-day (Dougherty 2018; Hua et al. 2016; Sharrer et al. 2016; von Ahnen et al. 2018) with Weigelhofer and Hein (2015) reporting removal as high as 166 g phosphate-P/m^3^-day for straw-filled bioreactors. Dissolved P load and concentration reductions by woodchips across bioreactor literature have been as high as >50% (Dougherty 2018; Hua et al. 2016; Husk et al. 2018), though most reported reductions are more moderate (≈10%; Goodwin et al. 2015; Warneke et al. 2011; Zoski et al. 2013). While DRP removal has been observed across a variety of studies, P removal in woodchip bioreactors has not been systematically tested.

Because even low concentrations of highly bioavailable DRP can trigger eutrophication in freshwater, it is important to better understand DRP interactions within denitrifying woodchip bioreactors. Any P removal provided by this denitrifying technology is a “free” added value. Four metal elements present in wood, calcium (Ca), magnesium (Mg), aluminum (Al), and/or iron (Fe), capture P through precipitation and/or ligand exchange, depending on speciation (Penn et al. 2014; Penn et al. 2007). These four metals would each be present in bioreactor woodchip media, though in varying concentrations depending on tree species and environmental growth conditions (Koch 1985; Pettersen 1984). The primary objective of this study was to evaluate P sorption as a potential fate of DRP in woodchip bioreactors using small-scale batch testing with wood species containing a range of metal element compositions. It was hypothesized that woodchips containing relatively greater contents of Al, Fe, Ca, and/or Mg would provide greater DRP removal than woodchips containing lower concentrations of those elements.

Secondary objectives included systematically testing three additional factors (water matrix, particle size, and initial DRP concentration) to more deeply evaluate P dynamics associated with woodchips in denitrifying bioreactors. A more complex water matrix was tested by evaluating P-dosed river water (as a proxy for agricultural runoff and drainage) compared to P-dosed deionized (DI) water. It was hypothesized that if the batch tests were performed with river water, then greater DRP concentration reductions would be observed due to an addition of microbes, micronutrients, and salinity in the river water. Next, two woodchip particle size ranges (3.2–6.3 vs. 6.3–13 mm) were evaluated to test the hypothesis that greater DRP concentration reductions would be observed for smaller particle sizes due to the greater associated surface area. Lastly, it was hypothesized that a low initial concentration of 0.10 mg DRP/L compared to 1.0 mg DRP/L would result in a greater concentration reduction but lower overall mass removal. This followed work by Hua et al. (2016) who reported phosphate removal rates by cottonwood (*Populus* sp.) woodchips of 0.25 and 0.88 g P/m^3^-day at respective inflow concentrations of 1.0 and 10 mg P/L in column tests.

## Materials and methods

### Woodchip characterization and preparation

Seven wood types were selected based on general availability in the US Midwest region and a literature review of typical expected elemental contents (Fig. S1; Table S1). Cypress (*Taxodium distichum*) and white oak (*Quercus alba*) were obtained locally as live and deadfall branches (Monticello, IL, USA); hickory (*Carya* spp.) and poplar (*Populus* spp.) were obtained as untreated lumber from a home improvement store; and cedar (*Cedrus* spp.) and maple (*Acer* spp.) woodchips were obtained from a Midwestern supplier (Xylem Ltd, Cordova, IL, USA). Woodchips were also collected from a leftover pile at a full-size denitrifying bioreactor constructed in IL, USA, in October 2018 (“field bioreactor” chips) to provide a realistic comparison with woods of unknown origin. These woodchips were described by the contractor as an approximately 70/30 ratio of local hardwood/softwood (Forrest, IL, USA) that were originally double ground on a commercial-scale grinder.

The branches, lumber, and woodchips were all chipped with a residential chipper (Tazz 3” Chipper/Shredder, Earthquake brand, Cumberland, WI, USA), and sieved to two particle size ranges 3.2–6.3 mm and 6.3–13 mm. These size ranges were smaller than the 25 to 51 mm effective diameter recommended for use in denitrifying bioreactors (USDA NRCS 2020) but provided a sufficient woodchip supply within uniform size ranges after all were chipped on the residential chipper. Moisture content was performed by drying a sub-set of woodchips at 70°C until a constant mass was achieved. Total porosity and bulk density were determined in triplicate by filling glass jars with woodchips in layers, adding water, and weighing the jars after 24 h after they were topped up with water. Woodchip nutrient content analyses were performed using a wet digestion method (Table S1; Brookside Laboratories Inc., New Bremen, OH, USA) and metal element concentrations were measured by nitric acid-hydrogen peroxide digestion with quantification by inductively coupled plasma mass spectrometry (Institute of Environmental Sustainability, Loyola University of Chicago, IL, USA). Thus, we used total concentrations of Fe, Al, Ca, and Mg as proxies for potential P removal mechanisms, mediated by sorption via Fe and Al, and via precipitation by Ca and Mg. Though Fe and Al can engage in precipitation with P, and Ca and Mg can sorb P depending on speciation of these metal elements (e.g., calcium carbonates, iron oxides), for the purpose of explaining potential differences in P removal among woodchips, we relied on total concentrations of these metal elements (Penn et al. 2007). The woodchips were flushed with deionized (DI) water to avoid effects of bioreactor start-up P leaching and then air dried. The flushing was considered complete once woodchip outflow DRP concentrations were below the analytical detection limit of 0.01 mg P/L which took no more than 40 days (180 cumulative pore volumes).

Three of the woodchip types, poplar, white oak, and the field bioreactor woodchips, in the 3.2–6.3-mm particle size range, were investigated in a post hoc analysis using scanning electron microscopy (SEM) at the University of Illinois Beckman Institute for Advanced Science and Technology. The three woods were selected to give a range across P removal results and woodchip appearance (e.g., wood color). The woodchips were taken from pre-batch test supplies that had been stored air-dried for approximately a year; the specific woodchips used for the batch tests had been disposed of by the time this analysis was initiated. For the SEM, six woodchips of each type were placed on three separate aluminum disks covered in carbon tape. The disks were sputter-coated with a thin layer of gold-palladium alloy for 70 s to make the samples electron-conductive for imaging with a Quanta FEG 450 scanning electron microscope (Thermo Fisher Scientific). Images were taken at ×60 to ×12000 magnification with 10kV beam voltage and a spot size of 3 nm for each of the woodchips.

### Batch experiments

Thirteen 72-h batch tests were performed with replication of four (Table 1). The first seven batch tests were conducted to compare individual wood species with varying elemental content of the same particle size (6.3–13 mm) with DI water dosed to 1.01±0.01 mg DRP/L. These first seven tests served as the controls for six additional tests which explored three independent factors: (1) water matrix: P-dosed DI water vs. P-dosed river water; (2) particle size: 3.2–6.3 vs. 6.3–13 mm; and (3) initial DRP concentration: 1.0 vs. 0.10 mg/L.

**Table 1:** Treatment descriptions of thirteen 72-h batch tests with dissolved reactive phosphorus (DRP) mean ± standard deviation concentration reduction and removal per mass dry woodchip. Negative DRP removal values indicate DRP leaching. Each test was performed in quadruplicate (*n* = 4)

Treatment combination				P removal at test end	
Test	Wood	Size	Initial conc. and source	Concentration reduction	mg DRP removed/kg woodchip
#	Common name	mm	mg P/L	%	
1	Poplar ^a^	6.3–13	1.0 DI	11 ± 3.2% *	2.0±0.6
2	White oak ^a^	6.3–13	1.0 DI	−7.2 ± 3.2% *	−1.2 ± 0.5
3	Hickory	6.3–13	1.0 DI	2.8 ± 1.2% *	0.4 ± 0.2
4	Cypress	6.3–13	1.0 DI	−6.1 ± 5.0% *	−1.0 ± 0.8
5	Field bioreactor ^a^	6.3–13	1.0 DI	84 ± 16% *	13 ± 2.5
6	Cedar ^a^	6.3–13	1.0 DI	3.5 ± 11%	0.6 ± 1.7
7	Maple	6.3–13	1.0 DI	7.3 ± 5.0% *	1.1 ± 0.8
8	Poplar ^a^	6.3–13	0.78 River	87 ± 3.2% *	12 ± 0.4
9	White oak	6.3–13	0.68 River	−36 ± 17% *	−4.1 ± 1.9
10	Hickory	6.3–13	0.61 River	−3.6 ± 7.9%	−0.3 ± 0.8
11	Poplar	3.2–6.3	1.0 DI	24 ± 2.2% *	3.8 ± 0.4
12	White oak	3.2–6.3	1.0 DI	4.3 ± 6.1%	0.8 ± 1.1
13	Poplar	6.3–13	0.10 DI	67 ± 4.8% *	1.2 ± 0.1

^a^A subset of batch solutions from one replicate of these tests were analyzed for metals and trace elements by inductively coupled plasma-atomic emission spectrometry

^*^Indicated the final test concentration mean at *t* = 72 h was significantly different (either lower or higher) from the initial test concentration (*ɑ* = 0.05)

For each test, 3 g of air-dried woodchips was added to 45 mL of either P-dosed (potassium phosphate monobasic, KH_2_PO_4_) DI water or river water in a polypropylene conical tube. Once corrected for moisture content, this resulted in a 16:1–18:1 liquid to solid ratio, loosely following Svensson et al. (2014) who used a ratio of 20:1 in sawdust batch leaching tests. The tubes were secured to a shaker table set at 200 rpm and eleven sample events spanned the 72 h (2 min, 10 min, 30 min, 1 h, 3 h, 6 h, 9 h, 24 h, 33 h, 48 h, and 72 h). Each tube was destructively harvested; that is, samples were not collected repeatedly over time from the same tube to avoid changing the liquid to solid ratio over time. Because each of the thirteen tests was performed in quadruplicate, 44 tubes were used for each test (11 sample events × replication of 4). The tests were performed at 21°C except for the river water treatment (see below). All water samples were filtered within 15 min of sample collection (0.45 μm membrane), frozen, and analyzed for DRP (Lachat Quickchem, method 10-115-01-1-A instruments, Loveland, CO, USA). Sample pH was measured within 24 h of collection (pH meter Fisher Scientific AE150, Waltham, MA, USA).

For the three river water treatments (Table 1; poplar, white oak, and hickory), water was sourced from the upper Embarras River near Urbana, IL, in winter (January 2020) and stored at 4 °C to minimize room temperature-associated changes in the water chemistry. These upper headwaters of the Embarras River are heavily dominated by tile drainage and runoff inputs; thus, this water was intended to be a proxy for the relatively more complex water matrix of tile drainage/agricultural runoff compared to DI water. The river water had an initial DRP concentration of 0.05±0.01 mg DRP/L and was dosed to 1.0 mg DRP/L using KH_2_PO_4_. However, the dosed river water was mistakenly not shaken before the batch test which resulted in the initial dosed river water samples having concentrations ranging from 0.61 to 0.78 mg DRP/L. For the particle size testing, two size ranges (3.2–6.3 vs. 6.3–13 mm for poplar and white oak; Table 1) were selected based on availability once the woods were chipped. The 3 g of woodchips used in both sets of tests entailed approximately 35 and 20 woodchips in the small (3.2–6.3 mm) and large (6.3–13 mm) particle size ranges, respectively. These size ranges were smaller than what would be used in a field-scale bioreactor (USDA NRCS 2020) but the residential chipper provided the most woodchips in these ranges. Finally, one test was performed with a low initial concentration of 0.10 mg DRP/L compared to the other tests’ initial concentration of 1.0 mg DRP/L (Table 1; poplar).

A subset of batch solutions was selected for additional post hoc trace elements analysis by inductively coupled plasma-atomic emission spectrometry (ICP; Illinois Water Survey, Champaign, IL, USA; US EPA Method 200.7). Due to analytical cost, samples from only five of the thirteen tests were analyzed (Table 1; tests #1, 2, 5, 6, and 8). These five were selected to give a range across wood types (tests #1, 2, 5, 6: poplar, white oak, field bioreactor, and cypress chips) and across water matrices (e.g., poplar tests #1 and #8). Also considering cost, only one replicate for the selected treatments and only the sample events at *t* = 0, 2 min, 10 min, 1 h, 24 h, and 72 h were analyzed. The water samples were stored frozen for approximately 9–12 months prior to this supplemental analysis.

### Statistical analyses

Shapiro-Wilk and Brown-Forsythe tests were used to evaluate normality and equality of variance assumptions, respectively (Sigma Plot version 14.0). The seven wood types were compared using the non-parametric Kruskal-Wallis one-way analysis of variance test (ANOVA; *α* = 0.05) and then further evaluated using pairwise multiple comparison Tukey tests. For comparisons of discrete categorical variables (e.g., DI vs. river water) and changes in DRP concentration over time (initial vs. final concentration), Student *t* tests were used when assumptions of normality and equal variance were met. Mann-Whitney Rank-Sum test was used when the normality assumption was not met, and Welch’s *t* test was used when the assumption of equal variances was not met.

## Results

### Elemental and microscopy analyses of wood types

**Fig. 1:**
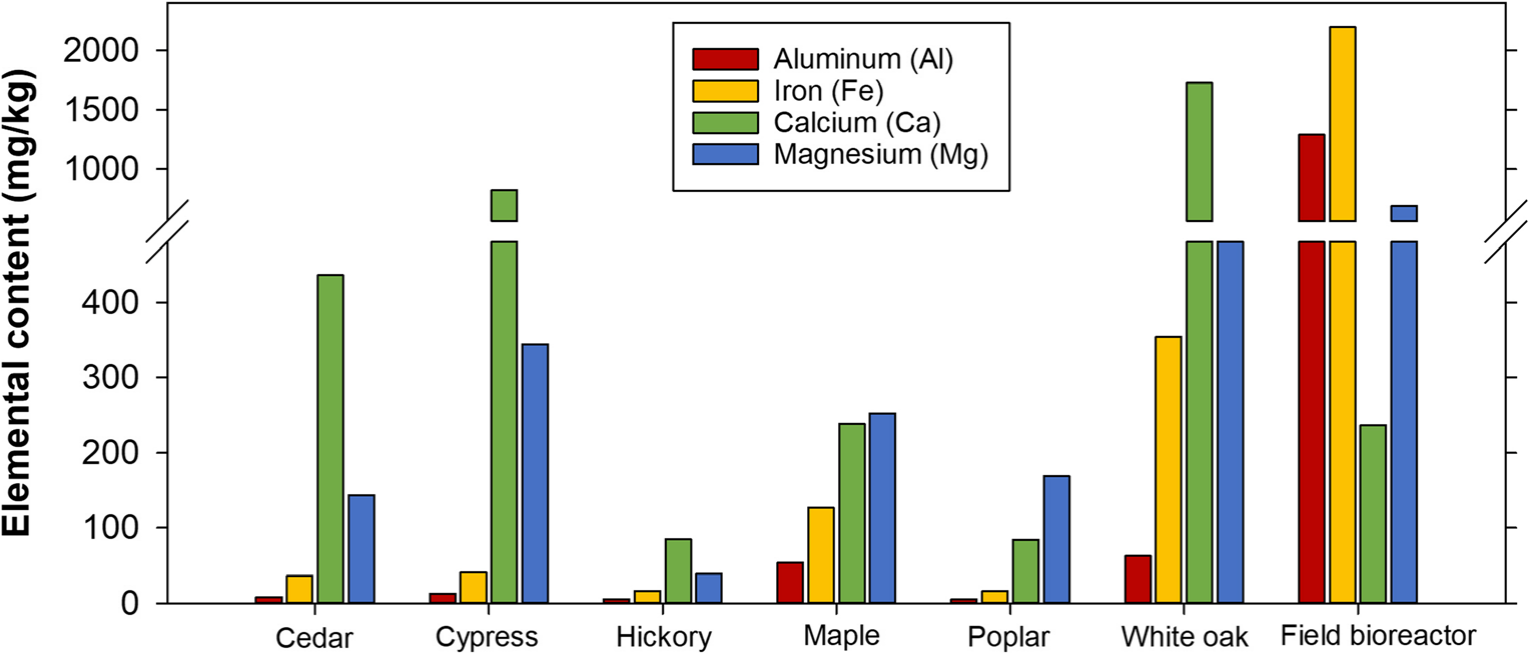
Elemental content for relevant phosphorus-sorption-related metals for seven wood types used in batch tests

The woodchips sourced from a surplus pile at a field bioreactor installation site had notably higher Al, Fe, and Mg, but not Ca, contents compared to the other woods of specific species (Fig. 1). Visual observation showed that they were darker than the other woodchips possibly indicating that there was soil mixed in with these woodchips (Fig. S1). The white oak woodchips also had relatively high values for these elements and contained the highest calcium content of the seven treatments by nearly twice (1720 mg Ca/kg). The poplar and hickory woodchips were relatively low in all four elements. These total metal contents allowed an initial assessment and comparison between wood types, although it is recognized that the potential reactivity of P sorbing media may be better characterized by amorphous Fe or Al oxides or water-soluble Ca (Penn et al. 2007; Qin et al. 2018).

**Fig. 2:**
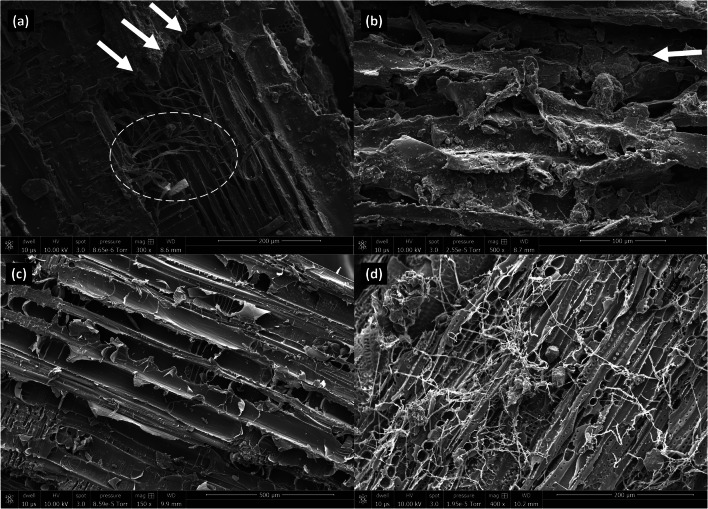
Scanning electron microscopy (SEM) images of initial field bioreactor (**a**, ×300; **b**, ×500), poplar (**c**, ×150), and white oak (**d**, ×400) woodchips prior to their use in batch tests. The arrows indicate degraded and broken edges and the dashed circle highlights fungal hyphae, both on the field bioreactor woodchips

The darker color of the field bioreactor woodchips was additionally explored using scanning electron microscopy which showed these woodchips to be relatively dirty and degraded (Fig. 2a and b). Some fungal hyphae were visible (Fig. 2a, white-dashed circle around spaghetti-like strings) but the more notable feature of these woodchips was their broken edges (Fig. 2a and b, arrows) and degradation of vascular structures. In contrast, the poplar and oak woodchips still exhibited tracheid and vessel cylindrical elements (Fig. 2c and d). The oak woodchips were widely covered with fungal mycelia (Fig. 2d, mass of white strings) much more so than the other two wood types on which scanning electron microscopy was performed. The chipped poplar lumber was sold as untreated lumber, but it was devoid of fungus and no bacteria were observed. Of the three types, bacteria were primarily observed on the white oak woodchips (Fig. S2).

### Batch tests

#### Wood types

**Fig. 3:**
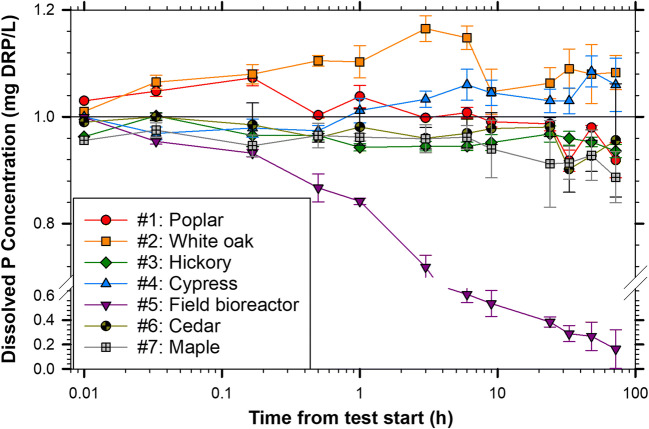
Mean ± standard deviation dissolved reactive phosphorus (DRP) concentrations for seven wood types in 72-h batch tests (*n* = 4). Test # in the legend refers to the test numbers in Table 1. The initial sample event at *t* = 0 was set at 0.01 h due to the logarithmic *x*-axis

Six of the seven wood types exhibited a significant change in batch solution DRP concentration over the 72-h test with four of those six providing 2.8–84% reductions in DRP concentration (field bioreactor, poplar, maple, hickory; Table 1, Fig. 3). The 84% DRP concentration reduction by the field bioreactor woodchips was significantly greater than the concentration changes caused by the white oak and cypress (*p* = 0.006 and 0.005, respectively, for pairwise multiple comparison Tukey tests) and was not significantly different from the four other treatments. The net DRP concentration changes for the poplar, white oak, hickory, cypress, cedar, and maple ranged from 11% reduction to 7.2% leached (Table 1) but were not significantly different from each other (*p* values ranging from 0.234 to 1.0 for pairwise Tukey tests).

**Fig. 4:**
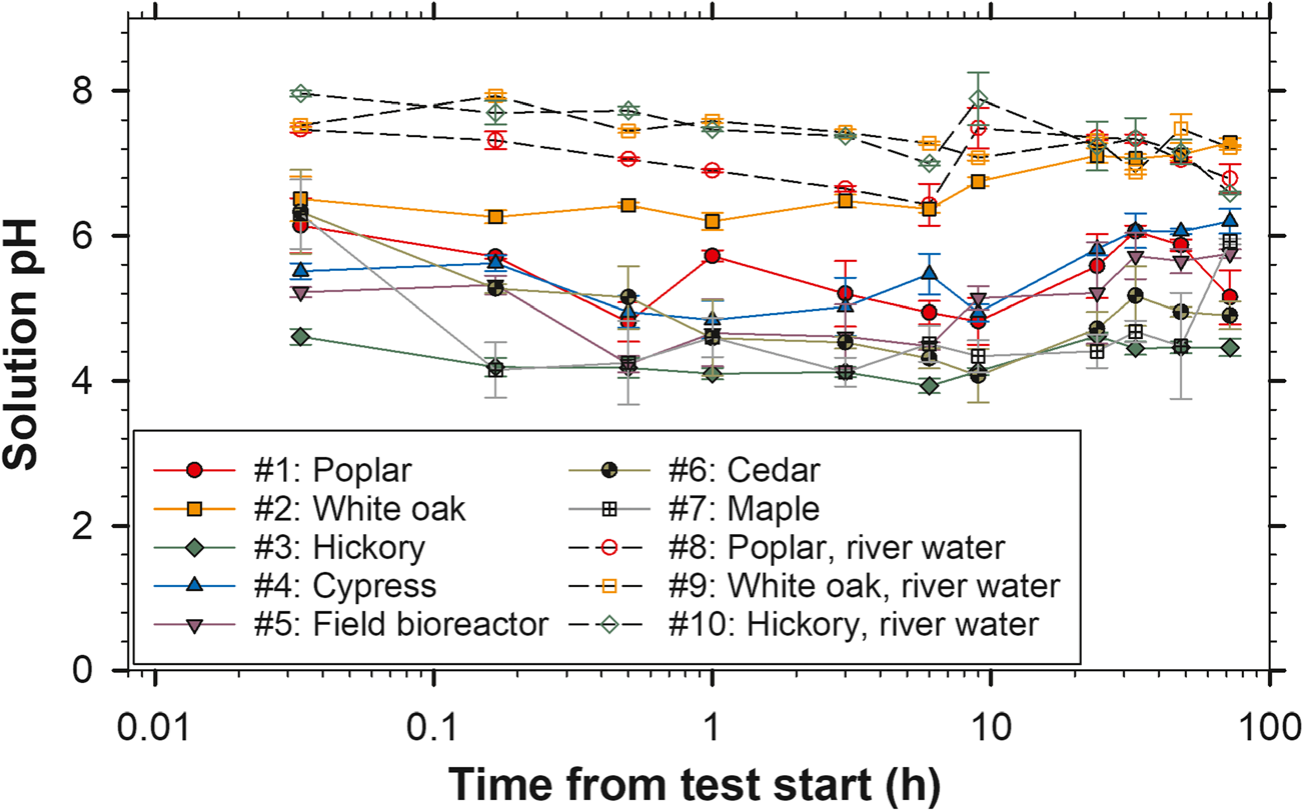
Mean ± standard deviation batch solution pH at each sample event for ten 72-h batch tests where P-dosed deionized and P-dosed river water were used (*n* = 4). Test # in the legend refers to the test numbers in Table 1

Cypress and white oak increased solution DRP concentration. In addition to having relatively high concentrations of metals known to sorb P (Fig. 1), the white oak also had a high P content of 0.035% compared to the other woods which ranged from <0.01 to 0.031 %P (Table S1). Most of the batch solution samples of these first seven tests had pH values below 6.0 (Fig. 4). The white oak leachate, which increased from pH of 6.51 to 7.29 by the end of the 72-h test, was the notable exception. The deionized tap in the lab provided water at a pH of 5.73 ± 0.07 and low values similar to this study (< pH 6.0) have been previously reported in woody media batch studies (Díaz-García et al. 2020; McLaughlan and Al-Mashaqbeh 2009). Cedar was the only wood type of the initial seven that did not significantly change the batch solution DRP concentration over the 72 h (Table 1; based on Welch’s *t* test comparing concentrations at initial and final sample events; *p* = 0.555).

**Fig. 5:**
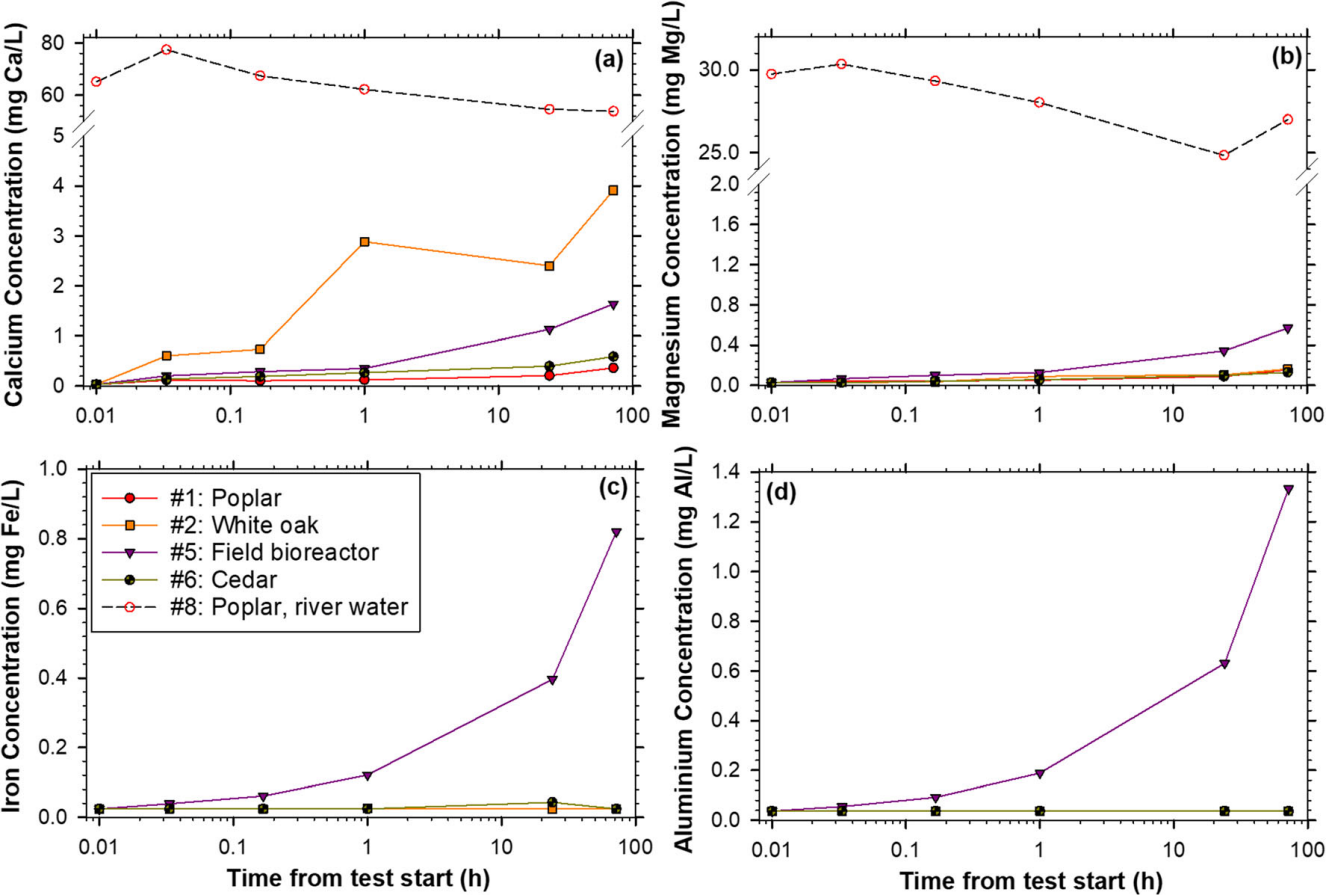
Calcium (**a**), magnesium (**b**), iron (**c**), and aluminum (**d**) concentrations for five treatments tested during 72-h batch tests (*n* = 1, only one replicate was analyzed). Test # in the legend refers to the test numbers in Table 1. *t* = 0 was set at 0.01 h due to the logarithmic *x*-axis. Note the *y*-axis breaks in panels **a** and **b**

There was a release of Ca into solution by the field bioreactor woodchips within 12 h and, even more notably, by the white oak within 1 h (Fig. 5a). The field bioreactor woodchips were the only treatment of the five tested using ICP that released Mg, Al, or Fe into the batch leachate (Figs. 5b–d).

#### Water matrix: deionized vs. river water

**Fig. 6:**
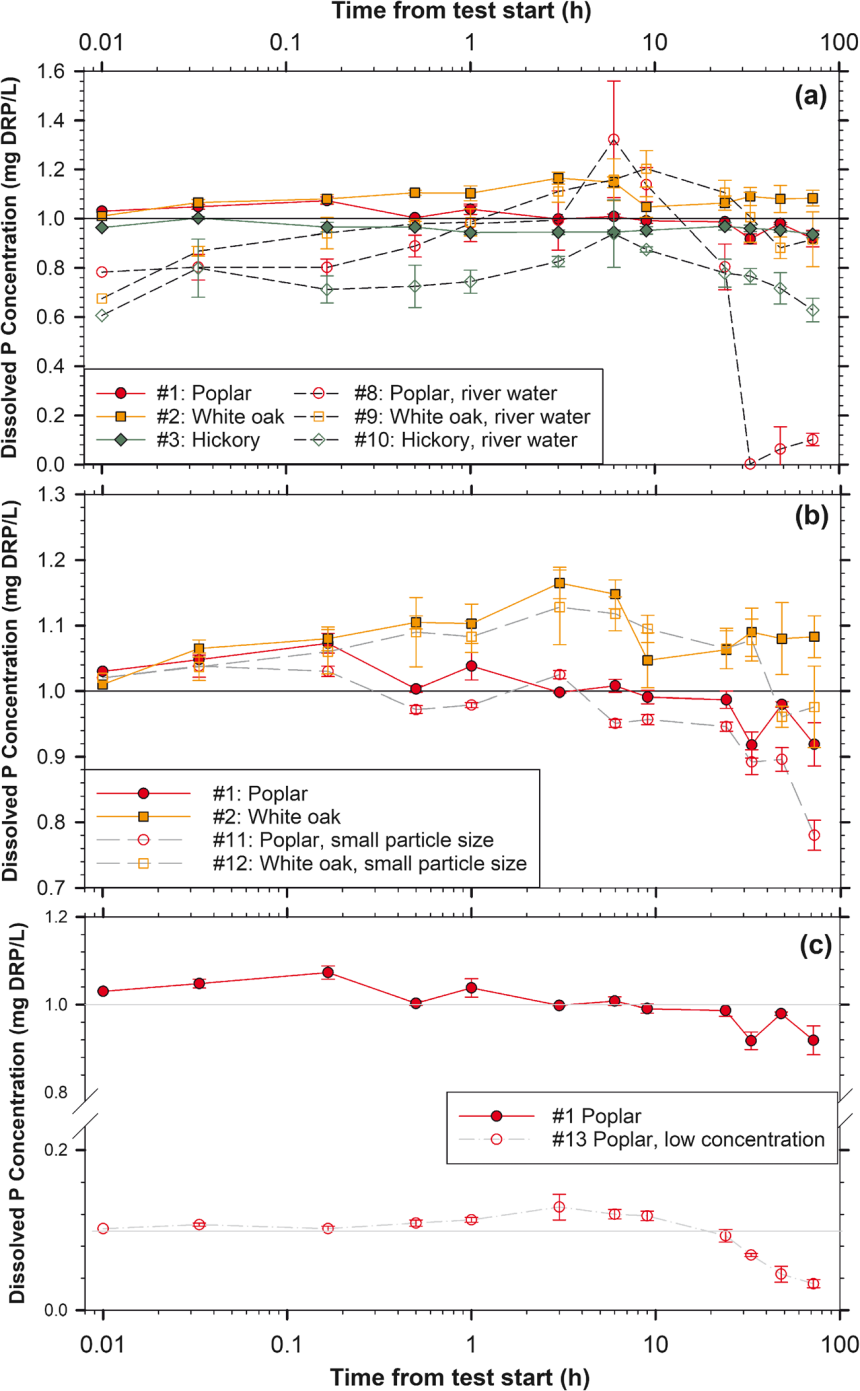
Mean ± standard deviation dissolved reactive phosphorus (DRP) concentrations for testing with P-dosed deionized water vs. P-dosed river water (**a**), 3.2–6.3 (small) vs. 6.3–13 mm woodchip particle size (**b**), and low vs. high (0.10 vs. 1.0 mg DRP/L) initial concentrations over 72-h batch tests (*n* = 4). Test # in the legends refers to the test numbers in Table 1. *t* = 0 was set at 0.01 h due to the logarithmic *x*-axis. The differences between treatment means were not significant for the river vs. DI treatment (*p* = 0.583); significant for the small vs. larger woodchip size (*p* = 0.050); and significant for the low vs. high initial P concentration (*p* < 0.001)

Changes in DRP concentrations were similar for batch tests performed with P-dosed river water compared to P-dosed DI water (*p* = 0.583; river and DI treatment means; 16 ± 55% and 2.1 ± 8.0%, respectively). However, this comparison was confounded by differences in both water temperature and initial P concentration. Since the relatively cool river water (4 °C) acclimated to room temperature (20 °C) within approximately 3 h, it is possible that microbial processes may have been initially slowed, but subsequent warming contributed to the observed decrease in DRP later in the tests. The second difference was because the river water treatments were mistakenly not shaken prior to the start of testing, and the initial concentrations for those three river water treatments ranged from 0.61 to 0.78 mg DRP/L instead of the correctly calculated target of 1.0 mg DRP/L (Fig. 6a). Solution DRP concentrations increased for all the river water treatments until 6 to 9 h, at which point DRP concentrations declined. If concentration reductions assessed over the entire test were calculated using the correct 1.0 mg DRP/L as the initial concentration, the poplar, white oak, and hickory would have provided 90 ± 2.6, 12 ± 11, and 39 ± 4.6% DRP concentration reductions, respectively, rather than the 87 ± 3.2, −36 ± 17, and −3.6 ± 7.9% shown in Table 1. Assuming the correct initial concentration (after mixing) would have resulted in a significant difference between the river- and DI-dosed treatments (*p* < 0.001; river and DI treatment means, 47 ± 34% and 2.1 ± 8.0%, respectively).

#### Particle size: 3.2–6.3 vs. 6.3–13 mm woodchips

Across wood types, smaller woodchips removed sixfold more DRP compared to larger woodchips, with mean reductions in DRP concentration of 14 ± 11 vs. 1.8 ± 10%, respectively (*p* = 0.050; Fig. 6b). Both chemical sorption and microbial immobilization mechanisms of P removal can be functions of surface area. Regardless, the smaller woodchip size tested here is not recommended for full-size bioreactor applications due to possible restriction of water flow (Van Driel et al. 2006).

#### Low and high P-dosed initial concentration

The lower initial concentration of 0.10 mg DRP/L resulted in significantly greater DRP concentration reductions than the higher initial concentration of 1.0 mg DRP/L (67 and 11% DRP removal, respectively, *p* < 0.001; Table 1; Fig. 6c). However, when expressed on a woodchip mass removal basis, the higher dosing test resulted in 2.0 ± 0.6 mg P removed per kg woodchip compared to the lower dose test for which only 1.2 ± 0.1 mg/kg was removed.

## Discussion

### Phosphorus removal by woodchips: proof of concept

The field bioreactor woodchips provided the greatest DRP removal (13 ± 2.5 mg P removed/kg woodchip) and concentration reduction (84%) during the first seven tests where wood types were compared (Table 1). The Al and Fe contents of those woodchips were notably high (Fig. 1) which suggested that, to the extent that these are present in amorphous (hydr)oxide forms, sorption reactions rather than Ca/Mg-associated precipitation may have been the more dominant chemical removal mechanism for those chips. However, as we did not quantify contents of reactive Fe and Al (e.g., (hydr)oxide minerals), nor the proportion of total Ca and Mg in water soluble form, which would engage in precipitation reactions, full certainty in ascribing mechanisms of P removal are not possible. Additionally, ligand exchange sorption reactions are relatively fast (e.g., < 20 min; Penn et al. 2007; Stoner et al. 2012), and the field bioreactor woodchips reduced the DRP concentrations consistently over the entire 72-h test. Such increasing DRP removal over this timeframe could have been because there was an abundance of P sorption sites which were never saturated. The possibility of microbial P removal (i.e., microbial growth which requires phosphate; Hua et al. 2016) also existed especially considering when these chips could have been relatively more inoculated given their source. However, bacteria were not widely detected on these woodchips using scanning electron microscopy and such removal predominantly occurs under aerobic conditions which would have been most likely early in the tests (dissolved oxygen and redox potential were not measured).

The solution pH provided additional evidence that if chemical P sorption was occurring, it would be associated with Al or Fe rather than Ca or Mg as most of the batch leachate samples of the first seven tests had pH values below 6.0 (Fig. 4; except the white oak treatment). Phosphorus precipitation with Ca and Mg materials is most effective at a pH range of 6.0 to 7.5, whereas Fe and Al-based P sorption is optimized under acidic conditions (Penn et al. 2007; Qin et al. 2018).

The field bioreactor woodchip’s leachate water chemistry containing Al and Fe reflected the elemental analysis of these woodchips (Fig. 1; Figs. 5c and d). Lindholm-Lehto et al. (2020) reported peak Al concentrations in woodchip (silver birch, *Betula pendula*) bioreactor outflows of 0.055 mg Al/L. Lepine et al. (2020) observed a higher peak concentration of 0.84 mg Al/L in column tests with maple (*Acer platanoides*) and ash (*Fraxinus americana*) woodchips. Though batch solution Al was below the detection limit of 0.037 mg/L for non-weathered (i.e., “raw”) woodchips, the field bioreactor woodchip leachate reached a maximum of 1.33 mg Al/L. Similar to Al, nearly all the batch solutions tested below the detection limit for Fe (< 0.024 mg/L), except the field bioreactor treatment which peaked at 0.82 mg/L. Rivas et al. (2020) reported an increase in Fe across a bioreactor in New Zealand treating dairy pasture drainage (inflow and outflow; 0.022 ± 0.019 and 0.219 ± 0.326 mg Fe/L, respectively). In the same year, there was 89% DRP removal across the bioreactor (inflow and outflow, 0.109 ± 0.195 and 0.011 ± 0.007 mg DRP/L). In the current study, the analysis of soluble metals in the batch solution, particularly for the field bioreactor woodchips, supported the possibility of DRP removal via chemical mechanisms.

The set of tests performed with dosed river water added more nuances to the possible sorption of DRP within a woodchip bioreactor. Calcium, magnesium (Fig. 5a and b), sodium, potassium, and sulfur (Figs. S3a–c; Table S2) concentrations were generally an order of magnitude higher in the one river water treatment compared to the tests where dosed DI water was used. Additionally, the river water batch tests had a higher mean solution pH compared to the DI tests (7.3 ± 0.37 vs. 4.9 ± 0.83; *n* = 33 and 153, respectively). The DRP mass removal achieved by the poplar woodchips in the dosed river water was the second greatest across all seventeen tests (12 ± 0.4 mg/kg; Table 1), even though the metal element content of the poplar woodchips was markedly low. It is possible that the relatively high metal element concentrations of the river water, which was used as a proxy for drainage water that would be treated in a bioreactor, masks the effect of lower concentrations of metal elements derived from the woodchips.

Water chemistry and microbiology are inexorably linked, and while the river water included naturally high concentrations of micronutrients, it also likely contained a microbial community different from the DI water. These river water tests were not designed to partition any observed DRP removal into sorption vs. microbial pathways, although microbial P uptake could have been possible. The relative possibility for and contribution of microbial P uptake vs. P sorption mechanisms in woodchip bioreactors is an area suggested for further research. Both wood media and water matrices are highly complex in this application in that while chemical sorption due to woodchip elemental content may be possible, it would likely be one of a variety of mechanisms at play in more real-world bioreactor settings.

### Wood composition and P removal

Wood elemental content varies based on factors such as tree species, age, growth conditions, and part of the tree (Koch 1985; Ovington 1959). In addition, the woods tested here spanned a variety of sources (lumber, fallen branches, commercially available chips) and types (softwood gymnosperms, hardwood angiosperms). While there is a range of what might be expected for elemental content of wood, the range across treatments presented a real-world scenario of the variability of woods available for bioreactors.

The wood elemental analysis and associated solution water chemistry of the field bioreactor woodchips may have been influenced by attached soil particles. Someshwar (1996) reported that soil could indeed become attached to wood (e.g., sand imbedded in bark). It was also possible that the brownish color of the field bioreactor woodchips may have been due to the decay of the wood or due to a brown rot fungus (Schwarze 2007). There was little evidence of active fungal colonization under the scanning electron microscope, but the broken and degraded vascular structures were notable on these chips compared to the other two viewed in this analysis (Fig. 2). Moreover, the field bioreactor woodchips were reportedly mainly hardwood (angiosperm) and brown rots are more associated with softwood species (gymnosperm, conifers; Schwarze 2007).

Beyond that unique field-sourced treatment, the chipped white oak deadfall branches had the highest Ca content (Fig. 1) and leached a notable amount of Ca into solution (Fig. 5a). Lindholm-Lehto et al. (2020) observed Ca concentrations in birch wood bioreactor outflows initially greater than 10 mg Ca/L which flushed to less than 5 mg Ca/L. This was in good agreement with the end of test concentrations here of 3.9 mg Ca/L from white oak, with concentrations ranging from 2 to 3 mg Ca/L between 1 and 24 h. Calcium and Mg must be released into solution for P precipitation to occur (Penn et al. 2007), and this timeframe (1 to 24 h) is a realistic bioreactor retention time indicating that it could be possible for Ca from woodchips to precipitate P in this application.

The use of oak woodchips in denitrifying bioreactors is restricted by the USDA NRCS Conservation Practice Standard due to this wood’s high tannin content which was assumed to negatively impact the denitrifying community and/or the downstream aquatic environment (USDA NRCS 2020). Despite this concern, oak wood may inherently support greater denitrification potential than other woods (Wickramarathne et al. 2021). Under the scanning electron microscope, the white oak presented a distinctive case of fungal colonization as nearly all the images showed large masses of hyphae (Fig. 2d). The uniqueness of oak in bioreactor applications was further confirmed here by the leachate Ca dynamics, proliferate fungal colonization, high solution pH (Fig.4), and DRP leaching even after pre-test flushing (Fig. 3). It is important to select woodchips for bioreactors to provide suitable N removal and also avoid pollution swapping.

The hickory and poplar woodchips were both chipped from store-bought lumber and both were considered to have relatively low metal cation content. Pettersen (1984) reported two poplar species contained 800–1200 mg Ca/kg and 270–290 mg Mg/kg; these values were much higher than the chipped poplar lumber used here which contained 84 mg Ca/kg and 169 mg Mg/kg. Nevertheless, the poplar woodchips resulted in DRP removal across nearly all tests (Table 1; 87, 67, 24, and 11% in four tests). Relatively consistent DRP removal by wood with comparatively low metal element content highlights that P removal is not reducible to woodchip composition alone and underscores the complexity of P-wood interactions specific to tree species.

### Scalability and application

Denitrifying woodchip bioreactors treating subsurface drainage water would generally be subjected to DRP concentrations much lower than the 1.0 mg DRP/L used as the initial concentration in most of the tests here. For example, the 25th and 75th percentiles of more than 400 site-years of drainage dissolved P concentrations were 0.016 and 0.064 mg DP/L, respectively, in a large-scale review by Hertzberger et al. (2019). Only one woodchip type was tested under the low concentration conditions (test #13; poplar with 0.10 mg DRP/L), but it was notable that the final DRP concentration achieved with this low range test was 0.033 ± 0.005 mg DRP/L (Fig. 6c). The relatively high percentage concentration reduction (67%, third highest of the 13 tests) and notable mass removal (1.2 ± 0.1 mg/kg) of this low range test supported the value of monitoring DRP dynamics at field-scale bioreactors, as subsurface drainage concentrations tend to be low but can be above values thought to lead to eutrophication (e.g., > 0.038 mg TP/L for lakes in the US Corn Belt and Northern Great Plains; USEPA 2002).

Using the range of observed mass removals of 0.4 to 13 mg DRP/kg and assuming an average bioreactor size of 100 m^3^ (following Christianson et al. 2021) and bulk density of 200 kg/m^3^ (e.g., Goodwin et al. 2015) would result in DRP removals of 8.0 to 260 g at the field scale. Assuming an average drainage area of 20 ha for this hypothetical bioreactor would result in P loss reductions 0.40 to 13 g DRP/ha, albeit this would be on a one-time basis and the ultimate fate of this P is still unclear. However, at least two field bioreactors have provided DRP removal in their second year of operation (that is, beyond the first year of operation; Dougherty 2018; Rivas et al. 2020) meaning that additional field studies and laboratory mechanistic studies may help further inform the extent of this potential benefit.

## Conclusion

The significant differences in DRP concentrations over the batch tests demonstrated the possibility for woodchips to influence P dynamics in a bioreactor, both positively and negatively. Any consistent DRP removal would be an important added value benefit of this denitrifying technology. Woodchips that were sourced from the field and contained the highest aluminum and iron content provided the most dissolved P removal, but woodchips with very low metal content provided the second highest removal when they were tested with P-dosed river water. While the amount and speciation of metals in P sorbing media are important, DRP removal will also be influenced by water chemistry (e.g., the water’s pH, buffering capacity). It is likely that any possible DRP removal by woodchips in a denitrifying bioreactor is not reducible to woodchip composition alone as there would be a variety of mechanisms at play in real-world bioreactor settings with complex water matrices. While bioreactor DRP removal is likely to be small in magnitude, observed P removals here combined with the relatively few technologies for mitigating dissolved P once it is in agricultural runoff and effluents and the relatively low concentrations of P known to cause eutrophication in freshwater makes any such contribution to P loss reduction important, especially when this is an added value.

## Supplementary information

ESM 1 (DOCX 2071 kb)

## Data Availability

The datasets used and/or analyzed during the current study are available from the corresponding author on reasonable request.
