# Supplementary material for: Phosphorus removal in denitrifying woodchip bioreactors varies by wood type and water chemistry
Source: Environ Sci Pollut Res Int. 2021 Aug 30;29(5):6733–43. doi: 10.1007/s11356-021-15835-w (PMC8763764; doi:10.1007/s11356-021-15835-w)
Supplement: Supplementary file 1 — (DOCX 2071 kb) [file 11356_2021_15835_MOESM1_ESM.docx]

Phosphorus removal in denitrifying woodchip bioreactors varies by wood type and water chemistry

Ana Paula Sanchez Bustamante Bailon^†^, Andrew Margenot^†^, Richard A. C. Cooke^‡^, and Laura E. Christianson^†^*

^†^ Department of Crop Sciences, University of Illinois at Urbana-Champaign, AW-101 Turner Hall, 1103 S. Goodwin Ave., Urbana, IL 61801, USA;

^‡^ Department of Agricultural and Biological Engineering, University of Illinois at Urbana-Champaign, 1304 W. Pennsylvania Ave., Urbana, IL 61801, USA.

* Corresponding author: [LEChris@illinois.edu](mailto:LEChris@illinois.edu); Ph. 1-217-244-6173

**Supporting Information**

**Pages: 5**

**Figures: 3**

**Tables: 2**


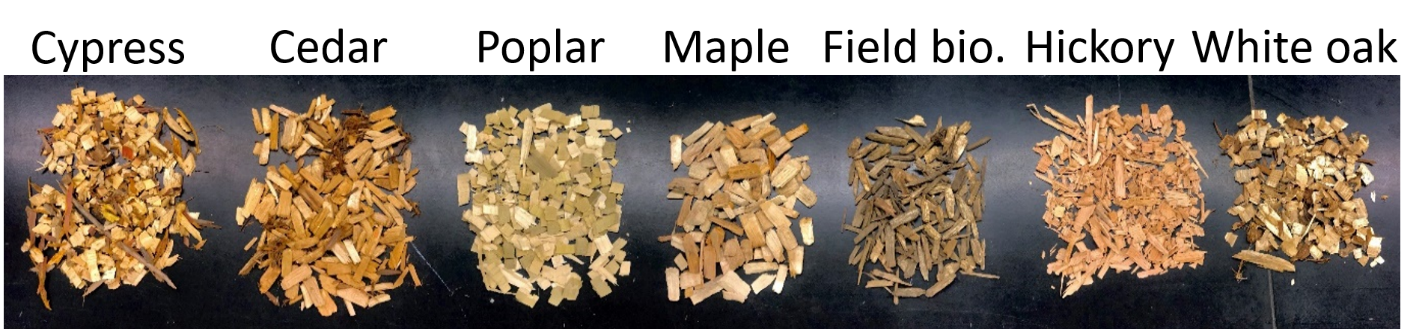
 **Fig. S1** Seven woodchip types sieved to the 6.3-13 mm particle size range for use in 72-h phosphorus sorption batch tests


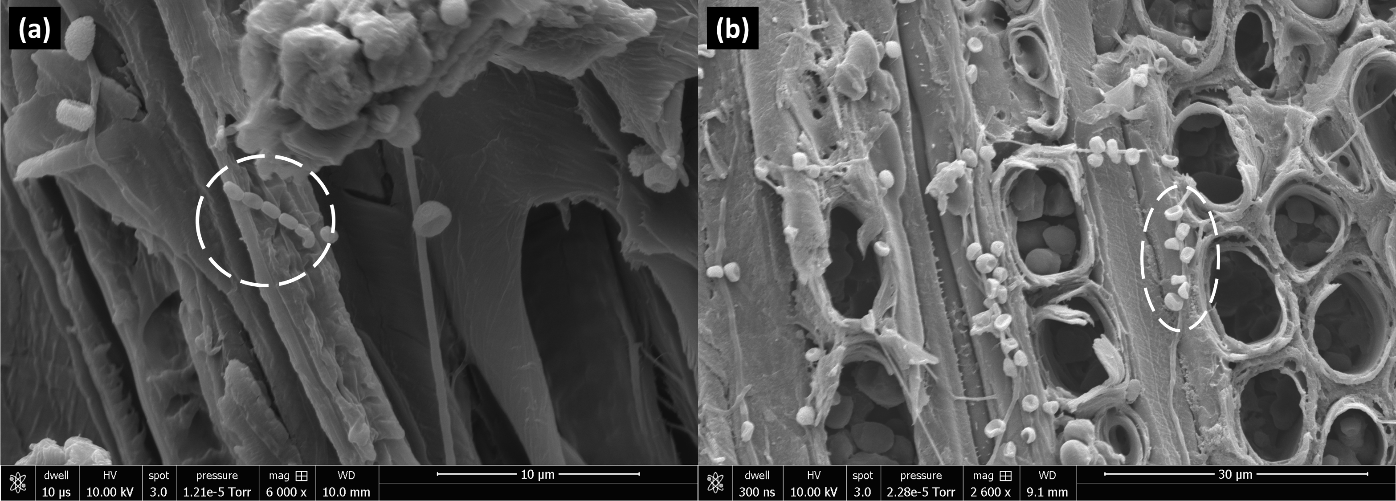


**Fig. S2** Scanning electron microscopy images of white oak woodchips prior to their use in batch tests showing bacteria (a, chain inside dashed circle, 6000x) and spores (b, cup-shaped features inside dashed oval, 2600x magnification)


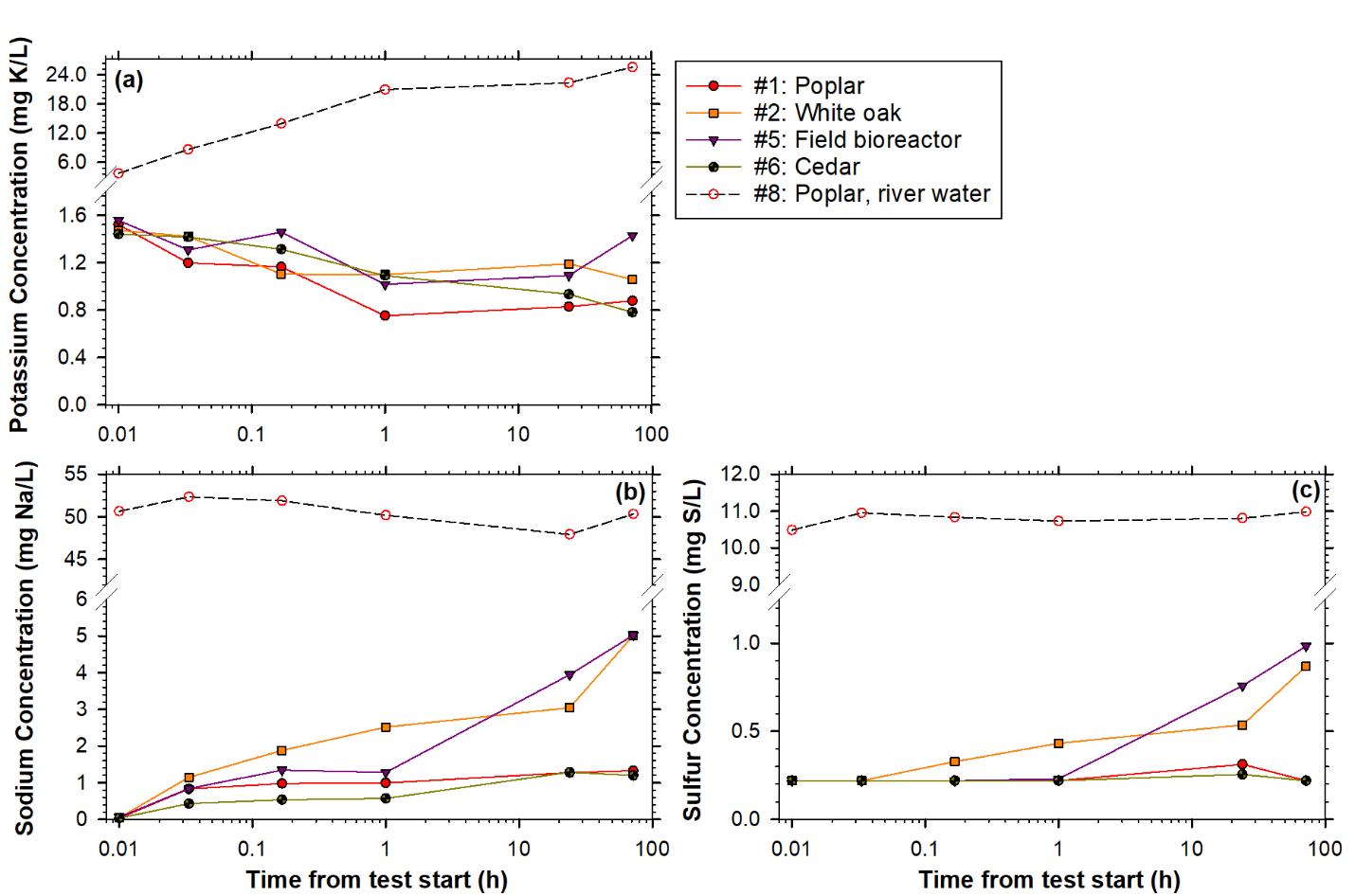


**Fig. S3** Potassium (a), sodium (b), and sulfur (c) concentrations for five treatments tested during 72-h batch tests (*n* = 1, only one replicate was analyzed). Test # in the legend refers to the test numbers in Table 1. *t* = 0 was set at 0.01 h due to the logarithmic x-axis. Note the y-axis breaks in each panel

Table S1 Physical characteristics and nutrient content of seven woodchips used in 72-h batch tests (C, carbon; N, nitrogen; P, phosphorus)

| Common name | Scientific name | Source | Bulk  Density | Total porosity | ------------- Nutrient content --------- | | | |
| --- | --- | --- | --- | --- | --- | --- | --- | --- |
|  |  |  |  |  | C | N | P | C:N  Ratio |
|  |  |  | kg/m^3^ | % | -------------- % ------------ | | |  |
| Poplar | *Populus spp.* | Lumber | 142 | 73 | 48.3 | 0.20 | < 0.010 | 241 |
| White oak | *Quercus alba* | Live branches | 191 | 74 | 47.0 | 0.39 | 0.035 | 121 |
| Hickory | *Carya spp.* | Lumber | 271 | 68 | 48.5 | < 0.20 | < 0.010 | 242 |
| Cypress | *Taxodium distichum* | Deadfall, live branches | 184 | 76 | 48.5 | 0.35 | 0.031 | 139 |
| Field bioreactor | Unknown, contractor sourced | Commercial woodchips | 227 | 68 | 47. 8 | 0.30 | 0.011 | 161 |
| Cedar | *Cedrus spp.* | Commercial woodchips | 152 | 69 | 49.9 | < 0.20 | 0.010 | 249 |
| Maple | *Acer spp.* | Commercial woodchips | 202 | 74 | 48.5 | 0.28 | 0.026 | 173 |

Table S2 Nutrient and elemental concentrations in the initial (*t* = 0) batch test sample from the poplar deionized (DI) water and poplar river water tests (tests #1 and #8) to compare the water chemistry. “IL WS” is the Illinois Water Survey; “UIUC” is the University of Illinois at Urbana-Champaign

|  | units | Analytical detection limit | Lab analyzed | Initial (*t* = 0) solution from: | |
| --- | --- | --- | --- | --- | --- |
|  |  |  |  | #1 Poplar, DI water | #8 Poplar, river water |
| Al | mg/L | 0.037 | ILWS | <0.037 | <0.037 |
| B | mg/L | 0.023 | ILWS | <0.023 | 0.119 |
| Ba | mg/L | 0.00085 | ILWS | <0.00086 | 0.059 |
| Ca | mg/L | 0.029 | ILWS | <0.029 | 65.13 |
| Cu | mg/L | 0.0016 | ILWS | 0.0025 | <0.0016 |
| Fe | mg/L | 0.024 | ILWS | <0.024 | <0.024 |
| K | mg/L | 0.016 | ILWS | 1.521 | 3.72 |
| Mg | mg/L | 0.027 | ILWS | <0.027 | 29.75 |
| Mn | mg/L | 0.0015 | ILWS | <0.0015 | 0.0547 |
| Na | mg/L | 0.036 | ILWS | <0.036 | 50.67 |
| S | mg/L | 0.22 | ILWS | <0.22 | 10.49 |
| Si | mg/L | 0.066 | ILWS | <0.067 | 4.190 |
| Sr | mg/L | 0.00037 | ILWS | <0.00037 | 0.118 |
| Ti | mg/L | 0.00056 | ILWS | <0.00057 | <0.00057 |
| Zn | mg/L | 0.0097 | ILWS | <0.0098 | <0.0098 |
| DRP | mg/L | 0.01 | UIUC Lab | 1.030 (dosed) | 0.783 (dosed) |
| NO_3_-N | mg/L | 0.10 | UIUC Lab | <0.10 | 5.00 |
